# Trichodentoosseous syndrome: a case report and review of literature

**DOI:** 10.1259/bjrcr.20190039

**Published:** 2019-11-15

**Authors:** Rohan Jagtap, Raghd Alansari, Axel Ruprecht, Deeba Kashtwari

**Affiliations:** 1 Department of Oral and Maxillofacial Diagnostic Sciences, University of Florida College of Dentistry, Gainesville, FL, USA

## Abstract

Trichodentoosseous (TDO) syndrome is a rare autosomal dominant condition characterized by various dental and non-dental findings such as taurodontism, amelogenesis imperfecta, osseous dysplasia, mandibular prognathism, curly hair, dysplastic nails, which may be symptomatic or asymptomatic. TDO syndrome is divided into three subtypes that helps to categorize different features seen in patients. There are very few cases reported in the literature of TDO syndrome. We present a case of a young adult male showing interesting Type I and II clinical and radiographic findings of the TDO syndrome. Amelogenesis imperfecta hypomaturation-hypoplastic type and TDO syndrome overlaps in their dental findings such as taurodontism and enamel hypoplasia and makes the diagnosis of TDO crucial. TDO syndrome was noted as an incidental finding on cone beam CT. This case report highlights the pathognomonic radiographic findings, treatment plan, and the clues to diagnosis this rare disorder. Management of TDO requires a proper diagnosis, multidisciplinary approach with comprehensive treatment plan including periodic follow up. Knowledge of this condition along with thorough interpretation of the entire cone beam CT volume are critical to understand this syndrome better due to its rarity.

In 1972, Lichtenstein coined the term “trichodentoosseous” (TDO) syndrome for a rare autosomal dominant disorder caused due to a mutation in the DLX3 gene on chromosome 17q21.^1,2^ According to the National Foundation of Ectodermal Dysplasia, some of TDO affected individuals inherit the mutated gene whereas others experience spontaneous gene mutation. TDO is subdivided into three types TDO I, II, and III based on clinical and radiographic features.^1^ Since this syndrome is rare, there is a limited number of the reported cases which makes it difficult to determine whether all reported findings, other than the pathognomonic ones, are part of TDO. Moreover, its rareness in the literature results in a lack of epidemiological data.^3^

TDO is characterized by abnormal development of ectodermally derived structures. Dysplastic nails, curly hair, bone sclerosis, taurodontism, and amelogenesis imperfecta are common features of this disorder.^1,3,4^ Additionally, some cases report maxillofacial findings such as mandibular prognathism, periapical abscesses, taurodontism, amelogenesis imperfecta, and impacted teeth.^4–6^ Of the other maxillofacial findings, taurodontism and amelogenesis imperfecta are consistently seen with TDO, whereas other non-dental abnormalities are variably, present. Some studies have even shown variability in non-dental features among the affected individuals who belong to the same family.^4^

The absence of the non-dental findings could confuse clinicians because TDO overlaps with amelogenesis imperfecta hypomaturation-hypoplastic type (AIHHT) in that both of them are characterized by taurodontism and enamel hypoplasia.^2^ However, the key factor to differentiate between them is that taurodontism associated with TDO is mostly confined to mandibular first permanent molars, whereas taurodontism associated with AIHHT could be seen in any molars.^7^

We present a classic case of TDO syndrome with florid osseous dysplasia (FOD) in the maxilla and mandible.

## Case presentation

A 22-year-old male presented to the University of Florida College of Dentistry, Oral and Maxillofacial Surgery clinic with a chief complaint of “I want to look at treating my jaw.” The patient reported a poor dentition his entire life, and he feels that his teeth have been “falling apart.” The remainder of the medical history was noncontributory. There was no relevant family history, and he denied a history of pain, numbness/paresthesia, facial asymmetry, or drainage. Intraoral examination revealed condyloma acuminatum on the lower lip.

**Figure 1.**
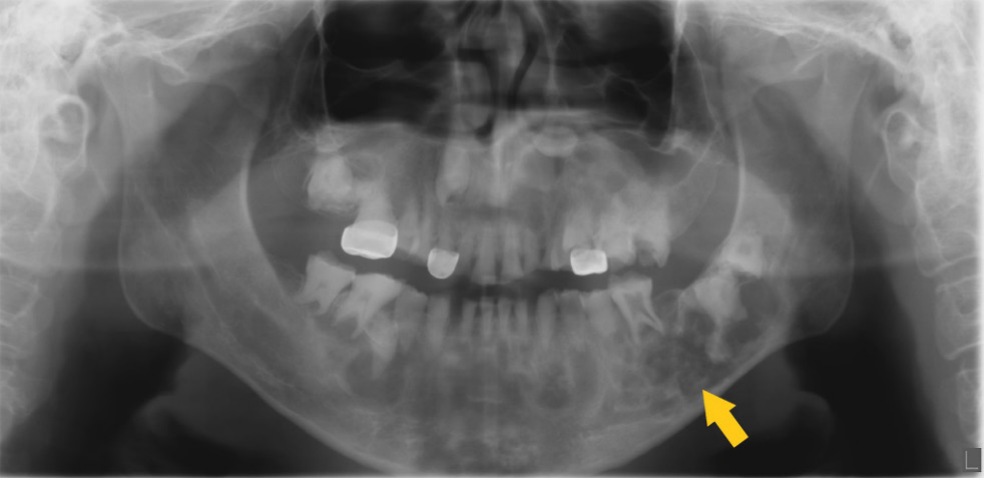
Panoramic view: depicting mixed density lesions in maxilla and mandible. The inferior alveolar canals are displaced inferiorly, especially on left side (yellow arrow).

A pantomograph was made as part of the diagnostic work-up which revealed mixed radiopaque lesions in the maxilla and mandible with multiple impacted and malformed teeth (Figure 1). Based on the clinical and radiographic findings, a cone beam CT (CBCT) volume was made.

**Figure 2.**
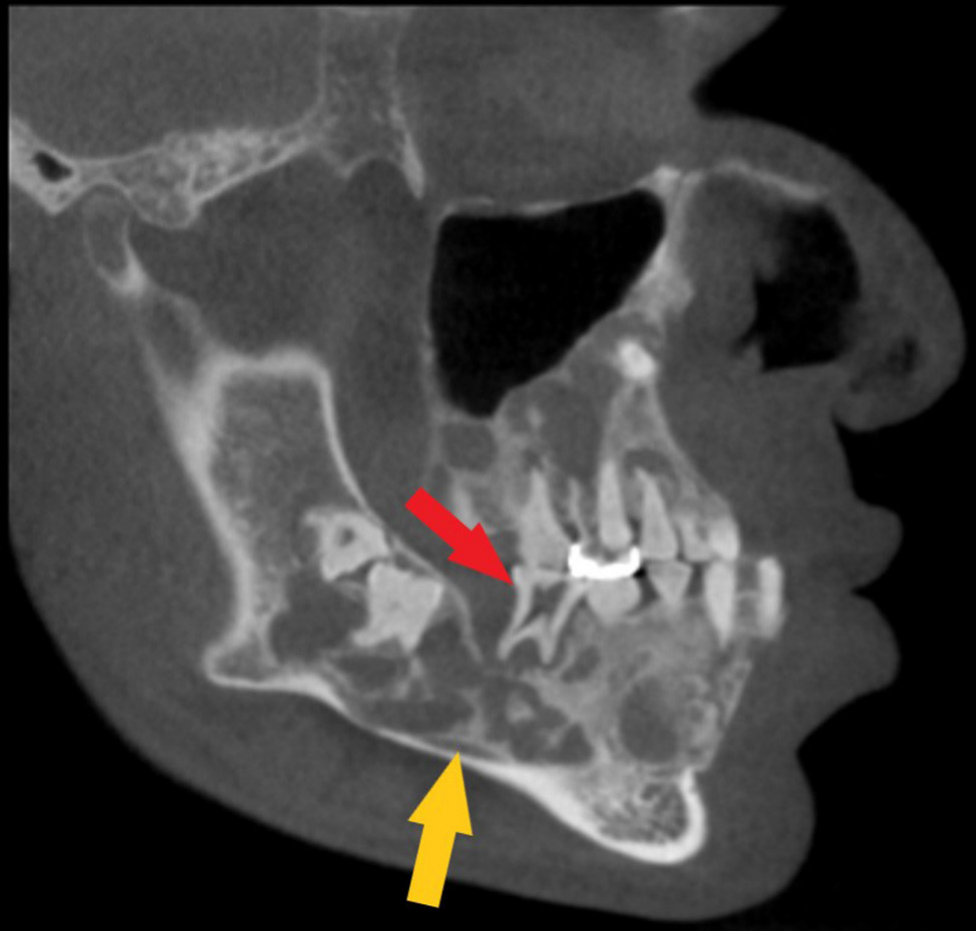
Sagittally reconstructed CBCT of the jaws depicting a mixed-density lesion in the maxilla and mandible containing teeth. There is elongated pulp chamber, apically positioned furcation, shortened roots are noted in tooth #19 and molars on right side, consistent with taurodontism (red arrow). Inferiorly displaced left inferior alveolar canal (yellow arrow). CBCT, cone beam CT.

**Figure 3.**
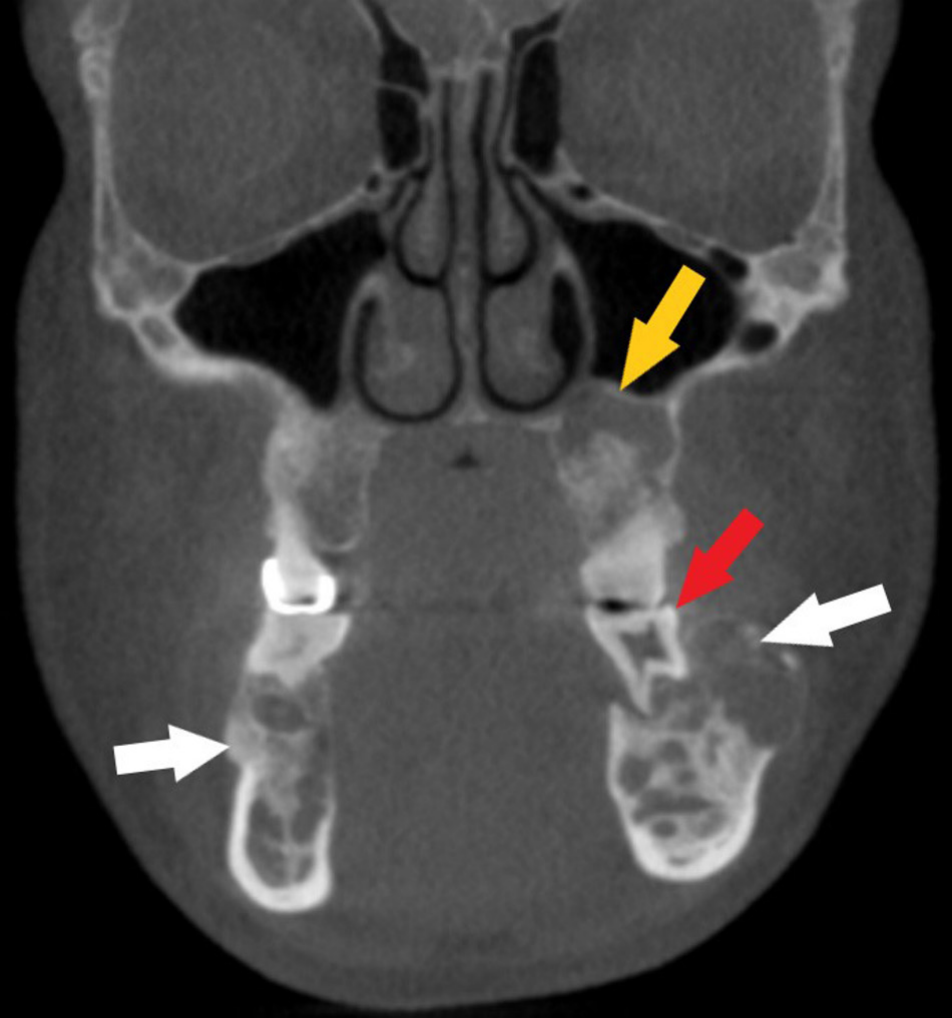
Coronally reconstructed CBCT of the jaws depicting a mixed lytic trabecular pattern and displacement of buccal cortical plate (white arrows). Taurodontism with shortened roots noted in #19 (red arrow). Superiorly displaced floor of the left maxillary sinus (yellow arrow). CBCT, cone beam CT.

**Figure 4.**
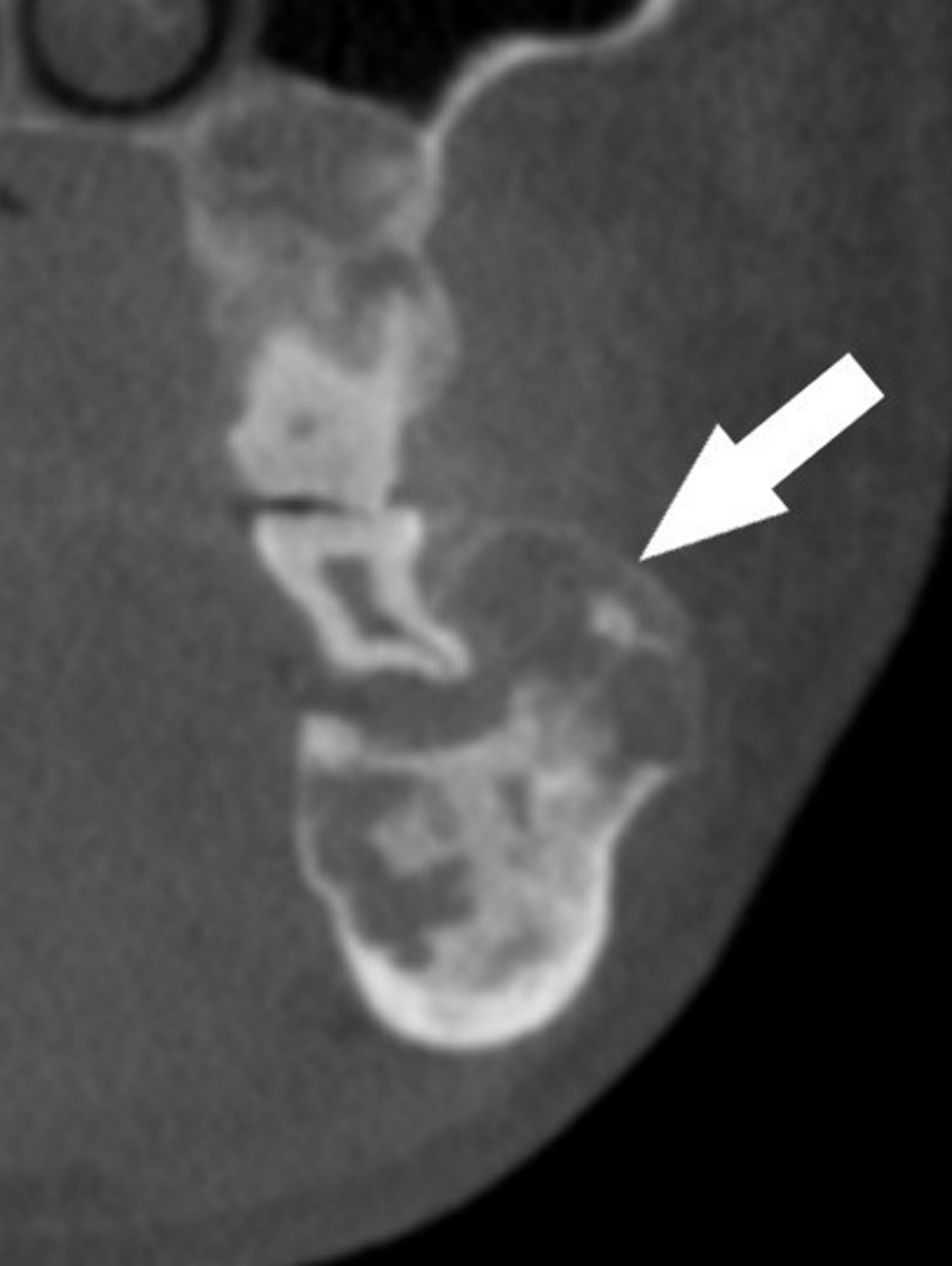
Coronally reconstructed CBCT of the left side of the mandible: depicting thinning of the buccal cortical plate. CBCT, cone beam CT.

**Figure 5.**
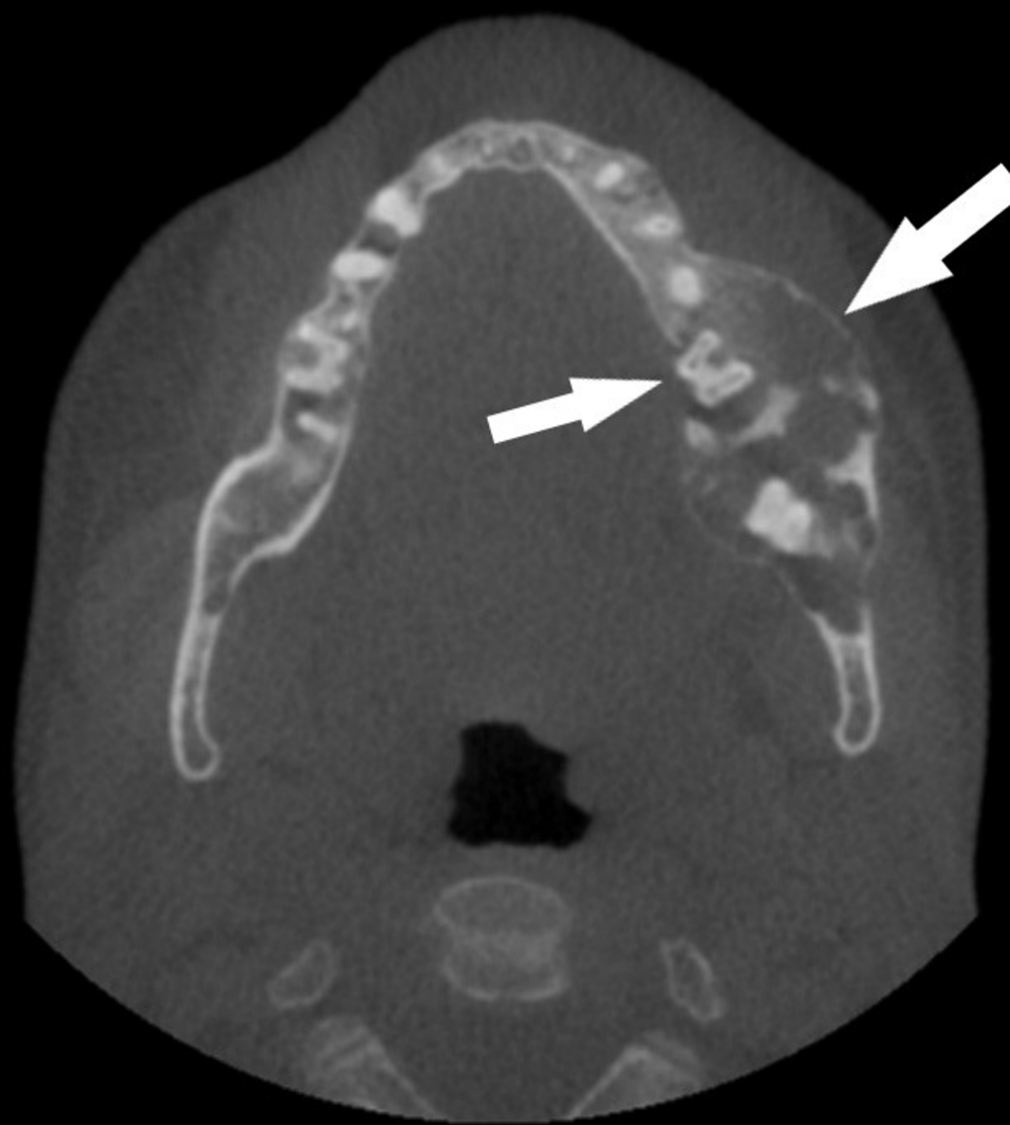
Axially reconstructed CBCT of the jaws depicting buccolingual enlargement and disruption of the lingual cortical plate. CBCT, cone beam CT.

The CBCT depicts a generalized multilocular mixed radiopaque appearance in the tooth-bearing regions of both jaws with the trabecular pattern having a mixed lytic and sclerotic appearance (Figures 1 and 2). There is absence of enamel in multiple maxillary and mandibular teeth (Figures 1 and 2). There is elongated pulp chamber, apically positioned furcation, shortened roots are noted in tooth #19 and molars of right side. The morphology of many of these teeth is consistent with taurodontism (Figure 2). There are multiple large radiolucent spaces within the bone (Figure 2). There is enlargement in several regions of the mandible and maxilla, especially in the left side of the mandible (Figure 3). There is thinning of the buccal cortical plate on the left side of the mandible and disruption of the lingual cortical plate (Figures 4 and 5). The radiographic interpretation is consistent with FOD with associated simple bone cysts, taurodontism, and amelogenesis imperfecta.

## Discussion

Jorgenson et al described dental abnormalities in TDO patients as dense bony cortex, amelogenesis imperfecta, and multiple impacted teeth.^8^ Our case reports the occurrence of four significant findings of TDO syndrome, *i.e.* FOD, amelogenesis imperfecta, taurodontism, and Class III malocclusion. Having all these lesions together in one patient is rare. FOD is a condition confined to the tooth-bearing regions of the jaw.^9^ It is considered a widespread form of a periapical osseous dysplasia, in which the dense osseous tissue in a background of fibrous connective tissue replaces normal cancellous bone.^10^ Various hypotheses have been postulated with regard to FOD’s pathogenesis, but the real cause remains unknown. Middle-aged black females are the most affected group. The bilateral posterior mandibular bodies are the most often reported sites, but the maxilla also can be affected.^9^ FOD is usually diagnosed as an incidental finding since this condition is mostly asymptomatic. The radiographic appearance varies from complete radiolucent through mixed (radiopaque-radiolucent) to complete radiopaque lesions.^9,10^ Treatment or intervention such as biopsy are not recommended in FOD patients, rather observation and periodic radiographic follow-up are advised. Avoidance of intervention is to obviate complications like prolonged poor healing and secondary infection which can lead to osteomyelitis or jaw fracture.^9^ The differential diagnosis of FOD includes Paget disease of the bone, chronic sclerotic osteomyelitis, and Gardener syndrome. Paget disease is commonly seen in white males, and has an increase in serum alkaline phosphatase levels.^9,10^ Radiographically, Paget disease affects the entire mandible whereas FOD affects the body of the mandible superior to the inferior alveolar canal. Chronic sclerotic osteomyelitis tends to be unilateral rather than bilateral, and it is not confined to the tooth-bearing region of the jaw. Gardner syndrome presents other skeletal changes such as osteomas, skin tumors or dental anomalies such as multiple odontomas, which is not the case in FOD.^9^

Amelogenesis imperfecta (AI), also known as congenital enamel hypoplasia, is a genetic abnormality that is characterized by abnormal enamel formation, which makes the teeth looks small, discolored, pitted or grooved, and prone to rapid wear and breakage.^6,11^ Both the deciduous and permanent dentitions can be affected. Researchers have found that the cause is a mutation in some genes that are responsible for protein encoding.^6^ This mutation can be inherited or due to a spontaneous gene mutation. AI is classified into 17 types based on the gene mutation pattern; however, in relation to clinical and radiographic appearance, there are four main types.^11^ First, hypoplastic AI type is characterized by thin, rough, pitted, and discolored enamel with lack of the normal proximal contour, radiographically the teeth appear to have square-shaped crowns, a thin radiopaque layer of enamel, and multiple open contacts.^6,10^ Second, hypomaturation type AI is characterized by soft, brown colored enamel of normal thickness, but with a radiopacity similar to dentin. Third, hypocalcified type AI is characterized by brittle, orange-brown colored enamel of normal thickness which has less radiopacity than dentin. Fourth, hypomaturation–hypoplastic with taurodontism type AI is characterized by thin, pitted, mottled, white–yellow–brown colored enamel which has a similar or greater radiopacity than dentin.^6^ Amelogenesis imperfecta can occur alone without any other signs and symptoms, or it can occur as part of a syndrome that affects multiple parts of the body.^6,11^

Taurodontism is a developmental dental anomaly that affects the morphology of molars producing wide pulp champers and, an apically positioned furcation thought to be due to disturbances in Hertwig's epithelial root sheath invagination.^12^ Taurodontism is most commonly found in the permanent dentition. It can be isolated, or as part of a syndrome such as Klinefelter, Down, or TDO syndrome.^13^ Radiographs are needed as the diagnostic tool that can be used to investigate a taurodont tooth as the external coronal morphology is within the range of normal.

As mentioned in the introduction TDO has three types. The first type shows the abnormal density of bone whereas the calvarium is within normal density. In addition, the patient displays sign of dolichocephaly due to early closure of the skull’s suture. Other features such as delayed teeth eruption, teeth discoloration, and malformed nails are seen in TDO-I. In the second type of TDO, calvarium exhibits osseous changes involving osseous dysplasia.^4^ Moreover, premature tooth eruption, curly hair, and dysplastic nails are observed among TDO-II affected individuals. In the third type, calvarium experiences changes in the thickness, however, the bone density is within normal range. Furthermore, macrocephaly is a marker sign of TDO-III.^1^ Our case demonstrates radiographic features from both TDO types I and II.

The rareness of TDO and limited reported cases are the main reasons that makes the agreement on specific signs debatable. Mandibular prognathism, dolichocephaly, periapical abscesses, and impacted teeth have been observed among the affected individuals.^4,5,13^ However, our case presents the reported radiographic features of enamel hypoplasia, first molar taurodontism, mandibular prognathism, impacted teeth, periapical abscess, and osseous dysplasia. Tight curly hair is one of the non-dental diagnostics findings, and indeed our patient has curly hair, but since our patient is an African American, we cannot attribute the hair curling to TDO. Taurodontism and enamel hypoplasia are the most common dental findings for TDO as well as for AIHHT.^2,6,7^ The confusion between these two conditions would be increased with the absence of the non-dental findings since there is variability in the presence of the non-dental findings. However, taurodontism which is associated with TDO is present only in mandibular first permanent molars which is not in case of AIHHT.^7^ Based on these markers, some researchers disagreed with certain reported cases diagnosed with AIHHT.^4,7^ For instance, the presence of taurodontism and curly hair in the family of the AIHHT reported case by Congleton and Burkesis led the researchers to believe that case is misdiagnosed as AIHHT instead of TDO.^4^ In our case, we have diagnosed the condition as TDO based on the pathognomonic radiological features.

The management of TDO affected individuals require a multidisciplinary approach involving both dentists and physicians. Periodic radiographic follow-up is required to prevent or long-term manage further complications such as osteomyelitis.^13^ In our case, FOD is one of the features that the patient has, which does not need any treatment unless it becomes secondarily infected, but it needs to be observed through periodic radiographic examination.^9^ Since TDO patients are prone to attrition, caries, abscesses and pulpal infections, prophylactic treatment and relieving pain play a primary role in TDO management.^13,14^ In addition, treatment of aesthetic defects and resultant psychological trauma are also important. Treatment of aesthetic defects resulting from amelogenesis imperfecta has shown to provide a marked increase in self-esteem of affected individuals as reported by Lindunger et.al.^15^ This restoration can be done through operative, prosthodontic, orthodontic, and/or endodontic intervention.^13^

## Conclusion

TDO syndrome is rare syndrome whose rareness has led to a lack of enough reported cases, which affects general knowledge about this syndrome. However, some signs have been found consistently in TDO affected individuals. Some of these signs also occur with AIHHT, but involvement of only the mandibular first permanent molar and a history of hair and nail defects could be used as distinguishing features. Genetic investigations can be helpful since the cause of this condition is a mutation in the DLX3 gene and could affect other family members. Treatment considerations are confined to each of the features that patients have. It is important to recognize multiple abnormalities associated with this syndrome radiographically. More reports of TDO syndrome with long-term follow-up information would help to understand this syndrome better.

## Learning points

1. TDO syndrome is rare autosomal dominant disorder.
2. Based on clinical and radiographic features, TDO is subdivided into three types TDO I, II, and III.
3. TDO is characterized by abnormal development of ectodermally derived structures with dysplastic nails, curly hair, bone sclerosis, taurodontism, and amelogenesis imperfecta are common features.
4. The absence of the non-dental findings could confuse clinicians as TDO overlaps with AIHHT that both of them are characterized by taurodontism and enamel hypoplasia.
5. Treatment considerations are confined to each of the features that patients have. It is important to recognize multiple abnormalities associated with this syndrome radiographically. More reports of TDO syndrome with long-term follow-up information would help to understand this syndrome better.
